# Supplementary material for: Identification of photoperiod-induced specific miRNAs in the adrenal glands of Sunite sheep (Ovis aries)
Source: Front Vet Sci. 2022 Jul 22;9:888207. doi: 10.3389/fvets.2022.888207 (PMC9354845; doi:10.3389/fvets.2022.888207)
Supplement: Supplementary file 1 [file Data_Sheet_1.ZIP › Supplementary file/miRDeep2 file.zip!/miRDeep2 file/Novel_Structure/SP42A3/Novel_356.pdf]

Provisional ID	:	NC_019467.2_15870
Score total	:	7.1
Score for star read(s)	:	3.9
Score for read counts	:	-0.3
Score for mfe	:	2
Score for randfold	:	1.6
Score for cons. seed	:	
Total read count	:	11
Mature read count	:	9
Loop read count	:	0
Star read count	:	2

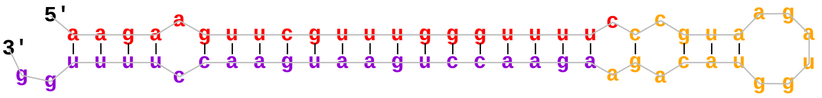

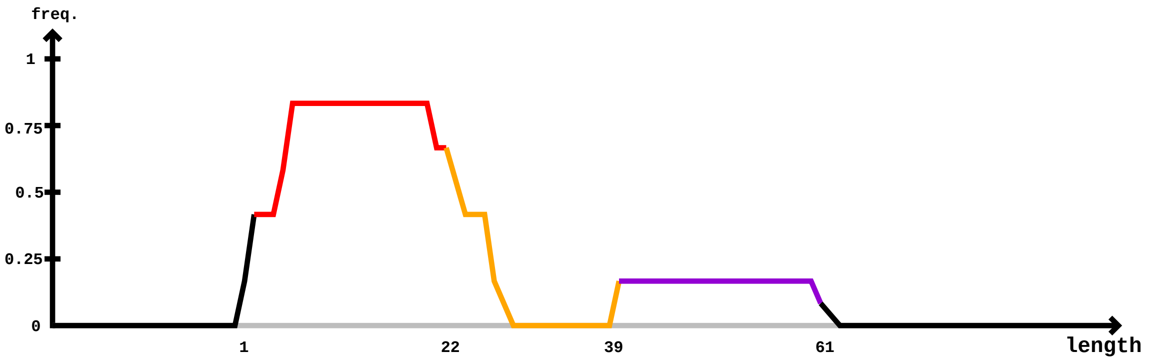

Mature Star

```
5'- acauaauauuggguggccaagaaguucguuuggguuuucccguaagaugguacagaagaaccugaaugaaccuuuuggccagcccaguagcugcugccucuuaaucuc  -3'  obs
    acauaauauuggguggccaagaaguucguuuggguuuucccguaagaugguacagaagaaccugaaugaaccuuuuggccagcccaguagcugcugccucuuaaucuc        exp
    ......((((((((.((((((((.(((((((((((((((.(.(((......))).).))))))))))))))).)))))))).))))))))((....))..........  reads  mm  sample
    .................caaAaaguucguuuggguuu.......................................................................  2      1   seq
    ..................aaAaaguucguuuggguuuuc.....................................................................  3      1   seq
    .....................aaguucguuuggguuuuccUgu.................................................................  2      1   seq
    ......................aguucguuuggguuuuccUgu.................................................................  1      1   seq
    ......................aguucguuuggguuuuccUgua................................................................  1      1   seq
    ......................aguucguuuggguuuuccUguaa...............................................................  1      1   seq
    ........................................................aAaaccugaaugaaccuuuug...............................  1      1   seq
    ........................................................agaaccugaaugaacUuuuugg..............................  1      1   seq
```
